# Vaccination concerns, beliefs and practices among Ukrainian migrants in Poland: a qualitative study

**DOI:** 10.1186/s12889-020-10105-9

**Published:** 2021-01-07

**Authors:** Maria Ganczak, Klaudia Bielecki, Marzena Drozd-Dąbrowska, Katarzyna Topczewska, Daniel Biesiada, Agnieszka Molas-Biesiada, Paulina Dubiel, Dermot Gorman

**Affiliations:** 1 Department of Infectious Diseases, Collegium Medicum, University of Zielona Gora, Zyty 28, 71-210 Zielona Gora, Poland; 2 Department of Public Health & Health Policy, NHS Lothian, 56 Canaan Lane, Edinburgh, EH10 4SG UK; 3 Primary Medicine Clinic, Parkowa 7, 74-100 Gryfino, Poland; 4 Pomeranian Medical University, Rybacka 1, 70-204 Szczecin, Poland; 5 Primary Medicine Clinic “Lancet”, Szkolna 9, 73-240 Bierzwnik, Poland

**Keywords:** Vaccination, Concerns, Beliefs, Practices, Migrants, Ukrainian, Poland

## Abstract

**Background:**

Ukrainians numbering approximately 1.2 million are the largest migrant group in Poland. Data on vaccination coverage among migrants are not collected in EU, including Poland. Therefore, this qualitative study aimed to identify vaccination practices in this migrant group, to explore facilitators and barriers to vaccination and related access to Polish healthcare services.

**Methods:**

In September 2019, a qualitative study of Ukrainian migrants (UMs) living in Szczecin, Poland, and recruited through a snowball sampling method, was conducted. Using a semi-structured topic guide, four focus groups were held with 22 UMs aged 18–45. Participants were asked about their attitudes towards vaccination in general with comparison between services in Poland and Ukraine. Following transcription and translation, a thematic analysis was conducted.

**Results:**

Respondents were distrustful of Ukrainian vaccination policy, medical personnel and individual vaccines, however, they often returned to Ukraine for dental and gynaecological appoint-ments. While critical with regards to registering with Polish GPs practices, UMs were confident in health professionals, as well as vaccine delivery. Vaccines were perceived as safer and of better quality than in Ukraine. Difficulties in translating vaccination records were rarely reported, verbal communi-cation was not problematic due to language similarities. All UM parents reported vaccinating their children according to the Polish schedule. However, a significant number of adult UMs have not completed mandatory vaccinations, although they may have obtained false immunization certificates; according to UMs those can be obtained by bribing. Participants reported lower acceptance of the influenza vaccine, mainly due to perceptions around its importance; none had been vaccinated against influenza. None of UMs had heard of the HPV vaccine. UMs experienced challenges in accessing credible online vaccination information in Ukrainian, no official local health authority vaccination material existed either, except for information about measles.

**Conclusions:**

This study pinpointed positive UM attitudes and practices regarding child vaccination in the Polish healthcare system and identified issues for improvement, such as adult vaccination. Health communication should be more tailored within UMs information delivery systems to enable migrants to make informed choices about vaccination. Further research is needed to better assess factors affecting vaccine uptake identified in this study.

**Supplementary Information:**

The online version contains supplementary material available at 10.1186/s12889-020-10105-9.

## Background

Since early 2014 there has been a rapid increase in migration to Poland driven by a strengthening economy. The largest group of migrants is from neighbouring Ukraine; 212,730 individuals according to the Statistical Yearbook of Poland 2019 [1, 2]. However, analyses conducted in 2018 on the basis of mobile telephony use indicated that the number of people from Ukraine residing in Poland was 1.25 million [3]. Ukrainian migrants (UMs) in Poland are typical economic migrants, predominantly working aged adults attracted by employment opportunities. In the early stages as they decide whether to stay, many fall into a pattern of working for several months in Poland and then returning to Ukraine to extend visas before again returning to Poland. Once settled in Poland travel to Ukraine remains common as many access healthcare there and their children have annual check-ups.

The literature highlights low vaccination coverage among migrants. Potential barriers include language or literacy limitations, financial and administrative problems, lack of knowledge about health and distorted perceptions about the risks posed by infectious diseases [4–8]. As well as different countries having different vaccination schedules and delivery mechanisms, migrants often originate from areas where access to routine immunization may have been interrupted [9].

During the Soviet era vaccination was centrally managed in Ukraine with high uptake [10]. However, the country has been affected by well-recognised political instability and economic challenges [9]. The healthcare system has been severely underfunded and the access to routine immunisation has been compromised. The United Nations International Children’s Emergency Fund (UNICEF) reported that although 90% of Ukrainian children were vaccinated against measles in 2018, however, only 60–69% against diphtheria and pertussis, and only 43% against polio [11]. In the World Health Organization’s (WHO’s) European region, Ukraine has reported the most measles cases: with 56,802, from 1.01–5.11.2019; a dramatic increase from only 39 cases in 2010 [12]. Notably, in Ukraine there is a culture reported where there is a black market in healthcare, based on informal payments and connections. There are reports of counterfeit vaccines and false immunization certificates which patients seek to avoid a perceived risk of vaccination [10].

In Poland, as in some other countries in eastern and central Europe, including Ukraine, mandatory vaccinations are included in the National Immunisation Programme and provided at no cost to all children, including immigrants. Even after the political system shift in 1989, mandatory vaccinations were maintained and widely accepted by Poles, assuring a high vaccine uptake [13, 14].

Even though the general population is well protected with adequate vaccination coverage and herd immunity, geographical clusters with high proportions of unvaccinated migrants can lead to outbreaks in host countries [6, 15–19]. Where UMs are concerned, there is a combination of a probably under immunised mobile population moving from a country with negative attitudes towards immunisation and high measles incidence [10, 12]. Thus it is not surprising that recent measles outbreaks related to UMs have been reported in Poland [20, 21]. In January 2019, the outbreak with 17 identified cases was reported in a 168-bed hospital in Szczecin, in the West Pomerania region. The index case was a Ukrainian hospital employee, who had returned from a visit to Ukraine 1 day before the onset of symptoms [21]. These outbreaks are evidence that vaccination programs do not reach all of those targeted by the vaccine, irrespective of origin country [19].

Data on vaccination coverage among migrants are not collected or monitored in European Union (EU) [6]. Similarly, there is no data on vaccination uptake among UMs in Poland. Therefore, this qualitative study aimed to identify vaccination practices in this migrant group, to explore facilitators and barriers to vaccination and related access to Polish healthcare services.

Qualitative research is particularly valuable for migration studies regarding its potential for producing comprehensive and refinement analyses adjusted for understanding the voices of immigrant groups. It facilitates conceptual modifications with higher validity, allows redefining the categories and creating new hypotheses, as well as exploring complex, multi-faceted dimensions of the migration process [22].

## Methods

### Study setting and population

A qualitative study was conducted in September 2019 in Szczecin, Poland. Four focus groups, with 7; 6; 4; 5 participants respectively, were held with Ukraine community members, recruited through a snowball sampling method in the Szczecin area with the inclusion criteria of age between 18 and 45 years, and been resident in Poland for a minimum of 6 months.

UMs were identified through general practices in Szczecin region and advertisements in Twitter and Facebook pages. Eligible UMs included parents and men and women belonging to the target groups for vaccinations, such as early child vaccinations regarding migrants’ children living in Poland and missing vaccinations (measles) in the adult population, as well as HPV and influenza vaccinations for adolescents/adults. All of the invited UMs were met at the Pomeranian Medical University in Szczecin, Poland.

Theoretical sampling was used, based on the emerging ideas in the analysis process as the criterion for additional data collection [23–26]. After the research topic and question were identified, the first group of UMs was invited to a focus group interview (Table 1). This was based on the demographic structure of the population of UMs in Poland [27] and included individuals representing adequate proportions regarding age, males/females ratio, participants with secondary/university degree, having/not having a child, in terms to sufficiently explore facilitators and barriers to vaccination and related access to Polish healthcare services. Following this initial focus group interview, the researchers met in a classroom at the Pomeranian Medical University and analysed the data. Based on the results from this round of data analysis, they identified more UMs to interview. As female migrants from the first group seemed to pattern their practices along their own, or close-relation’s experiences, whereas males provided more general comments in the first focus group, researchers decided to purposefully invite females and males in separate groups to further explore this theory. In addition, due to the fact that the first focus group comprised of UMs who already spent 2–4 years in Poland, the researchers decided to look for participants that have stayed in Poland for a shorter time to confirm/disconfirm previous findings, specifically about vaccination practices and access to Polish healthcare services; such participants formed the second focus group. An additional theory was that education level could shape opinions on vaccination. Therefore, the second focus group had been formed with participants having completed up to the secondary education. The researchers conducted interviews with that newly selected group of females and then analysed the data collected. Theoretical sampling continued like this, moving back-and-forth between sampling, data collection, and analysis, until the researchers reached the point of repetition of responses in the fourth group, and they felt they would fail to collect new information with subsequent group interviews [23–25].

**Table 1:** Characteristics of the participants, 2019; *n* = 22

No.	Code^**a**^	Age range (years)	Education level	Children	Duration of stay in Poland (years)	Back-and-forth travels ^**b**^	Reported vaccination refusals (participant/children) and vaccinations
GROUP 1							
1	9.1	31–40	Secondary education	Yes	4	1	Participant fully vaccinated in Ukraine aside from BCG vaccination declined. Children fully vaccinated in Ukraine.
2	2.2	31–40	Secondary education	Yes	4	1	Participant fully vaccinated in Ukraine aside from BCG vaccination declined. Children fully vaccinated in Ukraine aside from BCG vaccination declined.
3	8.1	18–20	Secondary education	Yes	2	1	Participant declined some early vaccinations in Ukraine. Child fully vaccinated in Poland.
4	6.1	21–30	University degree	No	3	1	Participant fully vaccinated in Ukraine.
5	5.1	21–30	University degree	No	3	1	Participant fully vaccinated in Ukraine.
6	1.2	21–30	Bachelor’s degree	No	3	1	Participant fully vaccinated in Ukraine.
7	6.2	31–40	University degree	Yes	4	1	Participant fully vaccinated in Ukraine. Child born in Ukraine, fully vaccinated there/in Poland.
GROUP 2							
8	7.1	21–30	Secondary education	No	2	1	Participant declined measles and hepatitis B vaccinations in Ukraine.
9	2.1	18–20	Secondary education	No	< 1	0	Participant fully vaccinated in Ukraine.
10	3.1	31–40	Secondary education	Yes	1	1	Participant fully vaccinated in Ukraine. Child born in Ukraine, lives in Poland, fully vaccinated in Ukraine.
11	1.1	21–30	Secondary education	No	1	1	Participant fully vaccinated in Ukraine.
12	10.1	21–30	Vocational education	No	< 1	3	Participant fully vaccinated in Ukraine.
13	4.1	31–40	Secondary education	Yes	< 1	1	Participant fully vaccinated in Ukraine. Children born in Ukraine, live in Poland, fully vaccinated in Ukraine.
GROUP 3							
14	5.2	21–30	Secondary education	No	2	1	Participant declined some early vaccinations in Ukraine.
15	11.2	21–30	Vocational education	No	< 1	0	Participant did not remember if declined some early vaccinations in Ukraine.
16	9.2	31–40	University degree	No	4	0	Participant declined some early vaccinations in Ukraine.
17	4.2	21–30	Secondary education	Yes	20	1	Participant fully vaccinated in Ukraine. Child fully vaccinated in Poland.
GROUP 4							
18	8.2	21–30	Secondary education	Yes	2.5	4	Participant declined some early vaccinations in Ukraine. Child born in Ukraine, still lives there, fully vaccinated.
19	7.2	> 40	Bachelor’s degree	Yes	< 1	1	Participant fully vaccinated in Ukraine. Children born in Ukraine, still live there, fully vaccinated.
20	12.2	31–40	Secondary education	Yes	2	2	Participant fully vaccinated in Ukraine. Child born in Ukraine, still lives there, did not receive some early vaccinations.
21	10.2	31–40	Vocational education	Yes	2	1	Participant declined some early vaccinations in Ukraine. Children born in Ukraine, still live there, and did not receive early vaccinations.
22	3.2	21–30	Vocational education	Yes	< 1	1	Participant fully vaccinated in Ukraine. Child born in Ukraine, still lives there, fully vaccinated.

^a^According to alphabetical order of participant’s initials

^b^Back-and-forth travels to Ukraine while staying in Poland

Participants were briefed prior to interviews; confidentiality was emphasised and that their insights into the Ukrainian community in general not only personal opinions were sought. Before the focus group, participants were asked to complete a consent form (in Ukrainian and Polish) and a short pro-forma about their socio-demographic profile (age, gender, education level, number of children), length of stay in Poland and vaccination status. Refreshments and transport to the meeting point was offered and each participant was compensated to the equivalent of £10.

### Data collection

An interview topic guide (Additional file 1*) was prepared by the research team following a literature review [4, 19, 28]. Topics included: general views about healthcare and experience of vaccination in Ukraine and Poland, sources of information about vaccination, vaccine quality and safety, risks posed by infectious diseases, access to healthcare in Poland, the availability of information from Polish health service workers.

The interviews were held in Polish, as the participants had a good command of this language. However, each participant was also given the translated written questions in Ukrainian. Focus groups were facilitated by two researchers: a physician, professor in epidemiology and infectious diseases at the Pomeranian Medical University (PUM), who had a clinical background, having worked as a doctor in an outpatient clinic, and by bilingual Polish and English researcher, MPH, information analyst at the Lothian Analytical Services, NHS Scotland. However, all other researchers actively participated in the focus groups discussions. They had academic research and/or clinical backgrounds. Such variety of backgrounds allowed to reduce potential desirability bias, regarding shaping of responds due to the role of facilitator.

The audio was recorded. Each focus group lasted approximately 1 h. Recordings were transcribed verbatim in Polish and then translated into English by the Polish researchers in consultation with a native speaker.

### Analysis

Thematic analysis was used as it offers an accessible and theoretically flexible approach to analysing qualitative data by searching for themes or patterns in relation to different epistemological and ontological positions [29]. The main question was “which approach to consider to make qualitative data, interpreted within a sociological epistemology, valid and relevant to the research objectives?”. Two research team members conducted a similar study among Polish migrants in Scotland [19] and brought their experience to the study regarding this question. Intensive literature searches, including both – textbooks chapters and articles on qualitative research methods [23–26], as well as articles on qualitative studies regarding attitudes towards vaccination [30–35], together with back-and-forth discussions via video-conferences until consensus could be achieved, helped researchers to proceed accurate qualitative analysis. This was performed with the help of the stages outlined by Braun and Clarke [29]: data familiarisation, coding and theme identification and refinement. All authors reviewed the translated transcripts independently and analysed their content assigning initial codes to text fragments. Afterwards the initial coding was compared, reviewed, discussed, and refined, which led to a more representative coding scheme and criteria. Then, codes were arranged in sub-themes and themes. The process of coding and development of themes was inductive in nature, as the authors had not planned beforehand to adhere to a pre-existing theoretical framework. Finally, the research team agreed on four key analysis themes:

- Vaccine delivery and PHC access\
- Trust in state structures, quality of healthcare and vaccination policies
- Trust in the vaccination provision
- Trust in the vaccines provided (safety/quality/importance)

## Results

Our focus groups contained 10 women and 12 men; one was 18 years, eleven were in their 20s, nine in their 30s, one in his 40s (Table 1). Seven had lived in Poland for under 1 year, fourteen 1–4 years and one for 20 years. Six held university degrees and twelve college qualifications. Seven participants had one child, five had two children (a total of 17 children with ages ranging from 6 months to 17 years); ten participants were childless. Participants had a variety of jobs; 6 women were cleaners at a teaching hospital, 4 worked as chefs in a restaurant, 3 were hospital maintenance workers, 3 - construction workers, 2 – packers, 2 – professional volleyball players, one – architect, one – laundry worker.

Eight participants stated that they had incomplete record of routine vaccinations. While one recalled her parents had refused to give her measles and hepatitis B vaccine, the group generally experienced difficulties in naming specific vaccines lacking in their immunisation history. Most of their children living in Poland were completely vaccinated according to the Polish schedule (two were not vaccinated with BCG). One UM reported having two children living in Ukraine who were completely unvaccinated.

### Factors affecting vaccine uptake, delivery and PHC access

#### Vaccine delivery and PHC access

Participants reported that it was straightforward and easy to book vaccination appointments for children at General Practitioner (GP) practices. In general, most UMs did not experience communication barriers during PHC consultations due to language similarities.

*“A GP asked about my child’s vaccination record. She did not understand what was written there, so she asked a senior doctor. They figured out that my child had been vaccinated correctly.”* (#4, Group 2)

However, some UMs reported institutional level difficulties in navigating the system, e.g. challenges in registering adult migrants with GPs practices with uncertainty about entitlement and proving residence.

*"For 4 days I was trying to get an appointment in the clinic, they did not have time to examine me. They kept saying “come the next day. We do not know what to do then, where to go.” (#6, G1)*

Those UMs that had experienced particularly high costs of paid health services in Poland (this mainly referred to dental and gynaecological services) and had quickly accessed much cheaper treatment on presentation to services in Ukraine:

*“I called him [a dentist] from Poland to make an appointment, to get in the queue, because it’s cheaper there. I go privately because the state-provided care is not any good.”* (#9, G3)

UM families were also reported to travel to Ukraine with schoolchildren for annual check-ups.

*“Either we or someone from the family travels with a child to Ukraine for an annual check-up*.” (#5, G1)

In general, all UMs reported difficulty finding vaccination information they trusted and resorted to using a variety of unregulated sources such as Google searches and social media, specifically Facebook.

*“Information about vaccines should be provided through the Internet, because everyone is on Facebook. Some advertisements need to be there.”* (#1, G2)

Participants advocated more vaccination information being available in Ukrainian language through different means.

*“A doctor would be the most trustworthy. He should cover a lot of information: what sort of vaccination is it, where it comes from, what side effects might be developed, etc… A doctor and, for example, a person who has already been vaccinated. Or a person who got sick due to the lack of vaccination.”* (#2, G2)

Regarding vaccine administration, it was also noticed by UMs that information about measles vaccination and antibody checking was widely distributed and available in Polish institutions which are obliged to serve Ukrainian citizens.

“*Information about measles vaccination was easy to find everywhere - at the Town Hall and the Provincial Migration Office. It was hanging on all doors. What is it, why is it important. In Polish, Ukrainian, even in English.” (#1, G1)*

#### Trust in state structures, quality of healthcare and vaccination policies

There was a higher level of trust in Polish healthcare system and vaccination policy compared to the Ukrainian one; this was partially shaped by reported differences in the quality of health services. The participants used the Ukrainian system as a point of reference.

UMs blamed the government and politicians for the long lasting healthcare crisis in Ukraine, pointing at rebuilding trust in the state institutions as the most important element to increase confidence in vaccinations.

Public employees were frequently viewed as corrupted and responsible for buying low quality vaccines which resulted in vaccine-related complications or even deaths.

*“They are buying a cheaper substitute for a vaccine, on this basis we do not really know what we get in this vaccine. Is there saline or something else?”* (#4, G3)

*“Many people do not believe what they are told about vaccines. My friends are afraid that they are carrying out experiments on us. They [the government] are crooks, they steal all the time.”* (#9, G3)

The commonly held view was that pharmaceutical companies promote specific vaccines to increase profits.

*“These vaccines are for the companies that produce them to make a lot of money, so people think.”* (#5, G3)

The high profile of vaccination in the media was well recognised with it frequently viewed as being important in fuelling fears and mistrust in vaccination.

*“It was blown out on television… showing children who died after vaccination.”* (#4, G3)

*“There was a programme where they said that there were some cases when a child dies. So people are afraid.”* (#5, G3)

Several participants contrasted the negative reporting in the media and were appreciative of the TV approach of showing the Ukrainian Minister of Health promoting child vaccinations and also vaccinating himself.

*“In one TV programme the Minister of Health said it [vaccination] was necessary and showed that he was doing it. He said that you had to vaccinate your children.” (#8, G4)*

#### Trust in the vaccination provision

UMs discussed between-country differences regarding vaccine administration. In Poland vaccines are administered by nurses, after a physical examination done by a doctor, while in Ukraine this depends on the region. In some regions the decision on whether a certain vaccination should be given was placed on the parent, who was asked whether a child was healthy; the vaccination is performed by nurses. In many other regions, like in commonly Poland, the patient is examined by a doctor before vaccination.

*“I think there are cases that people can die, but it can be, for example, the fault of the doctor – they do not carry out thorough examinations, they do not check the child's condition, although there might be some contraindications [for vaccination]. But in general, vaccines help people and they need to be given.”* (#9, G3)

*"In Ukraine when we went to the GP clinic with the child - the doctor did not carry out a thorough examination. The child could have been ill, though this seemed unimportant. The doctor said: “Do it today, because we do not know when the next vaccine supply will arrive.”* (#9, G1)

Additionally, Polish doctors were thought to be better and UMs were vocal and sceptical about the quality of medical training at some Ukrainian universities. Most UMs were not confident in Ukrainian health care workers (HCWs), however, they generally trusted Polish HCWs advice.

*“Everyone can be a doctor in Ukraine, because you may pay, then buy and have a diploma. I have more confidence in the doctors in Poland, they are trained, and they study medicine for several years.”* (#9, G3)

Lack of trust in healthcare professionals was dominant regarding access to all levels of services in Ukraine.

*“Where I live, when you go to the doctor, he does not see a sick person, he wants to get as much money as possible from you.”* (#7, G4)

*“They [doctors] say: Give us money and we will sell you a better product. If there is a thorough control over doctors, it would not be possible. But there is no control and money goes to them.”* (#10, G2)

Most UMs stated that in Ukraine false vaccination certificates can be readily obtained by bribing HCWs and were well known as a fact of life.

*“You can go to the GP clinic, to the hospital, give a bribe and they will write that all vaccinations have been completed and the child can go to school.”* (#10, G3)

*“If you pay you can get it [vaccination].”* (#9, G1)

#### Confidence in the vaccines provided (vaccination safety/quality/effectiveness/importance)

There was a uniform, strongly stated opinion expressed that the Ukrainian state-funded vaccines were inferior to those in Poland in many aspects.

*“I think vaccines are safer in Poland than in Ukraine.”* (#7, G1)

*“In the EU the quality of vaccines is better.”* (#8, G1)

*“Ukrainians don't trust vaccines. They think the vaccines are not safe and don't help.”* (#12, G4)

Respondents backed up comments about Polish vaccines being better with the evidence that family members were vaccinated in Poland while they had not been in Ukraine because of vaccine safety concerns.

*“My friend, an Ukrainian mother of a young child follows the vaccination program here in Poland. But she did not vaccinate her child while they were living in Ukraine. There are lots of mums like her.”* (#1, G2)

*“My brother received all vaccinations here, in Poland, he has not been vaccinated in Ukraine. He felt well after receiving vaccinations.”* (#2, G2)

One respondent presented clear evidence that medical staff may not be recommending state provided vaccines in Ukraine.

*“My friend took her neonate child to the vaccination unit. Her colleague who was a paediatrician and was working there, warned her not to immunize a child against poliomyelitis due to the poor quality of a vaccine. When my child was born I refused to vaccinate him with that vaccine. I told my GP the story I’d heard before. She did not vaccinate him, however she made a note that he was vaccinated.”* (#5, G1)

Some UMs stated confidently that in Ukraine vaccines could be falsely labelled and then administered under the public system.

*"They produce everything in Odessa, in Ukraine, but they can give vaccines false labels, like “produced in Germany” or “produced in England.”* (#9, G3)

*“We have a lot of black market vaccines.”*(#6, G1)

There were also concerns that some children had been vaccinated at clinics that experienced frequent power outages with no generator. Concern was raised that others had been vaccinated with vaccines which were past the expiration date or from a poorly stored multi-dose vial.

*“In our village three children aged about 1 year were given vaccines and died. The vaccines were poorly stored, and out of date. After that, everyone stopped vaccinating children.”* (#8, G1)

*“They keep vaccines in a regular fridge, in which a vaccine and a doctor's sandwich is kept.”* (#3, G2)

*“The vials are left opened after administration for 2-3 days after which other children are vaccinated with the same vial. A child could be vaccinated on the third day [*from the same vial]*, and could become sick. There have been many reports of this kind of behaviour. Therefore, there are refusals”.* (#9, G1)

UMs often sought branded vaccinations which were thought to be a “better choice”, although needing to be purchased by the parent.

*“A friend came for a vaccination to the doctor, they gave her child a bad vaccine and it was sick. But you could pay and the child would get a better vaccine.”* (#9, G1)

*“Good-quality vaccines are being sold for money.”* (#2, G2)

UMs mostly reported accepting mandatory vaccines according to the Polish schedule and generally thought that vaccines administered in Poland are safe.

*“I have two children, they live here. They were vaccinated here and they feel good. When my sister was at their age she was vaccinated, and then she was sick even more than before vaccination, my mother told me.”* (#1, G2)

*“Vaccinations are better here because there is a regulation.”(#11, G3)*

Some vaccines were considered less important than others and it was a common argument that there is no uptake of self-founded vaccines in Ukraine due to their costs, low salaries and difficult life conditions.

*“People in Ukraine do not earn enough, it is better to spend money on life. Everyone thinks what he will eat tomorrow, therefore, he does not think about [*self-founded*] vaccines at all.”* (#7, G4)

About a half of participants considered influenza vaccine important. However, this was the dominant vaccine that UMs reported struggling with the vaccination decision-making process, which involved the evaluation of perceived potential benefits and risks. Arguments against were mainly based on the perception that this vaccine is unnecessary. Influenza was considered less serious compared with other vaccine-preventable diseases (VPD). Some participants voiced higher concern around this non-mandatory vaccine that was considered not to be safe when administered in Ukraine.

*“I wouldn't get vaccinated against flu in Ukraine. It's better to take a lemon, I would figure out some natural things, it's safer than risking my health and have experiments made on me”.* (#1, G1)

It was also reported that no information, both on national and provincial level, was provided regarding influenza vaccination; no interventions oriented to high risk groups, e.g. pregnant women, to increase the uptake were provided either.

*“I was pregnant, I had the flu but I didn't hear about any vaccination from the doctor.”* (#8, G1)

Several UMs also reported concerns that having the vaccine during pregnancy could cause harm for a foetus.

None of participants was aware of the Human Papilloma Virus (HPV) vaccine, which has been widely available in Poland [25] but was not universally offered in Ukraine.

*“If I had heard about it [HPV vaccine] earlier, I might get my daughters vaccinated.”* (#2,G1)

*“I haven't heard anyone in Ukraine at school, on television talking about HPV vaccination.”* (#4, G2)

## Discussion

This is the first qualitative study asking UMs to Poland about vaccination attitudes and practices, as well as barriers and facilitators regarding immunisation. These were influenced by numerous interrelated factors, such as the lack of information on vaccinations, mistrust in Ukrainian governmental institutions and HCWs, perceptions of the quality, safety and importance of vaccines, as well as previous experience and current expectations around vaccination services and primary health care (PHC). Noteworthy, the female respondents patterned their practices along own or closed relation’s experiences, when males were providing more general comments.

### Vaccination uptake

UMs reported accepting and administering mandatory child vaccines according to the Polish schedule and generally thought that vaccines administered in Poland are safe and of good quality. Our results suggest that respondents cared much about completing mandatory vaccinations regarding their children living in Poland, however, they did not pay proper attention to their own vaccination status. More than one third of participants did not complete their mandatory child vaccinations. Factors which might influence poor vaccination coverage include their mistrust in the government and the health authorities and services which in turn shaped uncertainty in vaccine safety, quality and effectiveness. This was also reported by Ukrainian authors [36].

Migration from Ukraine has steadily grown in Poland, many of those migrants have not been vaccinated in childhood. However, some UMs obtained fake immunization records, and they travel back and forth for dental and gynaecological clinical appointments and children annual check-ups to their country of origin which is currently facing a measles epidemic crisis. Previous research also highlights that migrants may prefer to access health services in their country of origin due to lower cost and/or greater familiarity in their doctors [4, 28, 36–38]. Notably, several measles outbreaks have occurred recently in Poland and a dramatic (4-fold) increase of measles cases in 2019 when compared to 2018 [39]. In this context, it remains crucial, that Polish HCWs routinely explain to UMs the individual and population risk of not being vaccinated, as well as potential differences in vaccination delivery and scheduling. When obtaining and recording vaccination histories of UMs, Polish HCWs should be aware of the fact that some immunization certificates could be false.

Confusion arose for non-mandatory, self-paid vaccinations, which were reported rarely administered in Ukraine. Some vaccines, such as against influenza, HPV, rotavirus and varicella are recommended in the Polish immunization programme. However, their cost has to be covered by a patient. Participants reported that self-paid vaccines for children are not of interest for people living in Ukraine because such vaccines are perceived as too costly.

The same reason could have influenced UMs’ decisions regarding the completion of their own missing mandatory child vaccinations during their stay in Poland. Although trusting in Polish HCWs, as vaccination providers, as well as confident in vaccines quality and safety, they were willing rather to save money earned abroad then to spend any extra money to protect themselves from VPD. Different approaches to self-paid vaccines in Poland and Ukraine may additionally shape UMs opinions [40]. Despite economic reasons, the lack of information on vaccination benefits could also play a role [4, 19]. UMs complained that Ukrainians are not properly informed about the effectiveness and importance of self-paid vaccines. There are no sponsored vaccination programs for specific risk groups, such as senior citizens or pregnant women, either. Any vaccination in pregnancy was perceived as potentially risky and respondents clearly stated within Ukraine it is not recommended to vaccinate pregnant women.

The fact that the influenza and pertussis vaccine is offered during pregnancy in Poland, but not in Ukraine, may strengthen the impression that the Polish system is better equipped to prevent VPDs.

Interestingly, although none of UMs had been vaccinated against influenza in the last season and some UMs still held views that it is a relatively mild illness, more than a half were willing to vaccinate themselves against influenza while staying in Poland. Although none of our participants has heard about HPV vaccine, after obtaining adequate information during the focus group meeting, some would also consider it for their daughters, if the cost was refunded.

### Trust in national vaccination policies, vaccination providers and vaccines

Public trust forms a basis for vaccine confidence [41]. The Merriam-Webster Dictionary defines “trust” as assured reliance on the character, ability, strength, or truth of someone or something [42], while according to Russel [43] trust is an “encapsulated interest which fundamentally depends on perceptions of competence and motive”. In this context Larson et al. [41] state that vaccine confidence implies trust in those who make the decisions about vaccine provision (the policy-maker), trust in the vaccinator or other health professional (the provider), and trust in the vaccine (the product).

These trusting relationships are important to better understand the complex issue of vaccine confidence among UMs. Three type of mistrusts were reported in our study. First, participants pointed out problems related to reliance on the competence, and good faith of government authorities to recommend and furnish appropriate vaccines for Ukrainian citizens, as well as to provide medical professionals with adequate training programmes. Secondly, mistrust was directed towards local vaccination providers, concerning their qualifications, perceived corrupt practices, and safety of their vaccine storage and administration. Finally – trust in the product was seriously undermined and referred to manufacturers regarding effective, and uncontaminated, safe vaccines.

Participants trusted in the government-provided mandatory vaccination programme in Poland and it was praised for being free of charge. GP input within the child vaccination clinical pathway was reported as an advantage. Additionally, Polish doctors were perceived as better trained than their Ukrainian counterparts.

Ukrainian’s vaccination programme is very similar to that in Poland [44]. However, Ukraine introduced newer vaccines later than Poland, as an example, is yet to introduce pneumococcal vaccine, introduced in Poland in 2018 [45]. This could lead to UMs thinking their system is old-fashioned and not to the standard of the Polish system.

Since 2010, Ukrainian parents typically sign an informed consent form every time their child receives a vaccine [46], this is not practiced in Poland. Notably, such consent is not required by law. This developed as a medical community response to the risk of prosecution [36], which was also repeated by our participants. This could also reinforce the impression that the Ukrainian system is less patient friendly than the Polish.

About 60% of Ukrainian citizens state that to get adequate healthcare a patient has to bribe or use relevant connections [47]. In a nationally representative household survey, 57% of respondents reported they have ever personally paid informally in cash to physicians, medical staff or other personnel in health care facilities [48]. As much as 70% of patients make unofficial payments for health services they receive, including vaccinations [49]. This was confirmed by our participants: most argued that false vaccination certificates were available in Ukraine through bribery, this is mostly due to distrust in untrained physicians and universal fears about vaccine quality.

Participants clearly made a distinction between vaccine qualities in both countries. A belief was expressed that vaccines in Poland are modern, manufactured by well-known, trusted brands, properly stored and administered, where adverse reactions seemed to be much less common than compared to Ukraine.

There were also commonly held views among UMs that vaccines administered in Ukraine are falsely labelled, of doubtful quality and poor effectiveness and that vaccination in Ukraine could cause illness, even death. Almost all respondents mentioned paying for “better” child vaccines in Ukraine, in preference to the state-provided versions, with the main reason being to obtain a high quality product, and avoiding any adverse effects.

### PHC access and vaccine delivery

While critical regarding registering with Polish GPs practices, vaccine delivery was reported by UMs as patient-friendly process. Difficulties in translating vaccination records were rarely reported, although this was observed by other authors which surveyed migrants [4, 19]. Verbal communication or fulfilling vaccination schedule did not pose a challenge, possibly due to language similarities and minor variations between the national programs. However, UMs complained that there were no translated versions of vaccination materials accessible at the PHC facilities. This was also observed in other studies related to migrant populations [4, 7, 19, 23] and may reflect a lack of adequate information available for migrants. They have to rely on other sources for information about vaccines, mostly from the internet and social media, less commonly from friends or Ukrainian doctors. Of note, instead of advocating for improved immunization among their patients, Ukrainian doctors often have doubts about the importance of the immunization program [50].

Lack of knowledge about vaccines, fuelled by negligence of this subject by Ukrainian policy makers, HCPs and media make UMs vulnerable to accessing confusing, incorrect or biased information. UMs might form beliefs about vaccinations that have no scientific foundation. Furthermore, the anti-vaccination movement is large in Ukraine, for instance, organizing demonstrations against mandatory vaccinations [36]. The fact that our respondents mainly access Ukrainian sources of information, combined with the powerful anti-vaccination movement, may result in UMs in Poland being exposed to negative views about vaccination.

Subsequently, UMs tended to use informal networks, rather than professional help. UMs pointed at difficulties in communicating with Polish medical staff about the topic of vaccination. This finding corresponds with our previous research about vaccinations in various Polish population groups [51, 52] which found that HCWs are not fully providing information about vaccinations. Therefore, front-line HCWs should increase their role in sufficient information delivery to UMs regarding this important topic. This should be enhanced by providing UMs with translated versions of vaccination-related materials. This was also recommended by other literature [4, 8, 19].

### Study strengths and limitations

The strength of this study is its novelty. We have concentrated on UMs, the largest migrant group in Poland, but also on a group in which immunization coverage against most VPDs is much lower than in the general population. Furthermore, these migrants are travelling between Poland and a country in which the incidence of measles is one of the highest in the world [16]. To increase their vaccination coverage, it is necessary to identify their attitudes and practices regarding vaccination, as well as barriers and facilitators they experience in accessing Polish PHC services.

Another strength of this study is its potential for instrumental use in improving immunization activities targeting Ukrainian community in Poland.

The core weakness of this study is the relatively low sample size; the same issue was also discussed by other authors who conducted focus groups analyses [19, 23]. Although we believe that we reached data saturation in our sample, we are aware that other UMs, e.g. from other parts of the country, may have different experiences with vaccinations, as well as with the Polish health system. Holding more focus groups to improve saturation would be of value.

The researchers would expect the elderly UMs to think differently about vaccinations, this has been reported previously by other authors [1, 2]. However, this group of participants was considered not easily achievable, given practical considerations and the demographic structure of the population of UMs in Poland [3]. Furthermore, UMs in Poland are typical economic migrants, predominantly working-age adults, some of those living in Poland with children. Therefore, child immunisation and HPV vaccinations uptake among them and their children was our main point of interest. The authors consider the overall group structure adequate to gain insight into the topic.

A further weakness inherent to the focus group format is its participant snowball selection system — the results obtained are therefore harder to generalize to the larger population [49]. However, our participants differed regarding education level, job position and Ukrainian residence which meant relatively diversified opinions could be sourced.

The location where the interviews were held could have influenced respondents and a desirability bias could orient the overall trend of the results. However, the anonymity of the respondents could help to reduce this bias*.*

Furthermore, despite the best efforts of moderators, outspoken individuals could “hijack” a discussion in a focus group, meaning that other participants’ counter-views might be suppressed [53]. Still, this depends on the skill of the moderator. Having researchers from the National Health Service (NHS) Lothian, Scotland, who have already conducted similar studies among Polish migrants in the UK [19, 54, 55], was beneficial to facilitate the focus groups, as well as to analyse large volumes of qualitative data with Polish researchers and ensuring objectivity. In addition, the groups were made up of less than seven participants in any focus group to avoid hindering or restricting communication during the interview.

Finally, any opinions expressed in the four focus groups, although candid, could be also scientifically incorrect [19].

## Conclusions

The participants praised the Polish vaccine delivery system, thought that Polish doctors are well trained and the vaccines administered are safe and of good quality and also took their children for mandatory child vaccinations according to the Polish schedule.

However, adult UMs did not consider that their own vaccination status was of any importance. A significant number of UMs living in Poland have not completed mandatory vaccinations, although they may have obtained false immunization certificates. Additionally, they were not familiar and rather hesitant regarding self-paid vaccines. This is of particular relevance due to a rapid increase in Ukrainian migration to Poland, the fact that this population is not totally settled, and an ongoing measles outbreak in Ukraine.

The study also highlights the important unmet requests for information that UMs need to make informed vaccination choices, and the challenges of producing and delivering such information in a context of existing uncertainty. Combined professional information about vaccinations offered in Ukrainian, delivered not only by HCPs, but also with the use of booklets, web-based education and social media, could help address low uptake, especially in the light of the openness shown by UMs regarding this issue.

Further quantitative research is needed to better assess factors affecting vaccine uptake identified in this study.

## Supplementary Information

**Additional file 1.** Interview Guide. An English language version.

## Data Availability

The datasets used and/or analysed during the current study are available from the corresponding author on reasonable request.

## Abbreviations

EU: European Union
GP: General Practitioner
HCW: Health care worker
HPV: Human Papilloma Virus
NHS: National Health Service
PHC: Primary health care
UCM: Ukraine community members
UM: Ukrainian migrants
UNICEF: United Nations International Children’s Emergency Fund
VPD: Vaccine-preventable diseases
WHO: World Health Organization
