# Supplementary material for: Vaccination concerns, beliefs and practices among Ukrainian migrants in Poland: a qualitative study
Source: BMC Public Health. 2021 Jan 7;21:93. doi: 10.1186/s12889-020-10105-9 (PMC7789884; doi:10.1186/s12889-020-10105-9)
Supplement: Supplementary file 1 — Additional file 1. Interview Guide. An English language version. [file 12889_2020_10105_MOESM1_ESM.docx]

**INTERVIEW GUIDE**

**Qualitative study of vaccination views and attitudes among the Ukrainian community in Szczecin**

**English Version**

**I. Demographic Questions** (*used in a short questionnaire administered to all participants before the focus group discussion*)

1. Age: (how old are you) .....

2. Gender: Male / Female

3. Education: primary school / vocational school / high school / bachelor / university studies

4. Employment in Poland: public sector / private sector

5. Employment in Ukraine: public sector / private sector

6. Number of children (enter how many children you have) .....

7. How long have you been staying in Poland (please indicate the number of months or years) .....

8. How many times a year do you travel to Ukraine? ..... times

9. You describe your knowledge of the Polish language as: very good / good / poor / very poor

10. Your vaccination schedule - Do you remember what you were vaccinated for to date? Please, specify……………..

Do you remember any vaccination refusal? Please, specify......................

10a. For parents: Do you remember what your child was vaccinated for? Please, specify...............

Do you remember any vaccination refusal? Please, specify.....................................

II. **Topic Questions**:

**INFORMATION ON VACCINATION**

Do you think that there is sufficient information available in Ukrainian language about vaccinations?

Where do Ukrainians source information and advice about Ukrainian and the Polish vaccination programs?

How is vaccination mentioned in the state media?

How is vaccination mentioned in social media?

**PHC ACCESS AND VACCINE DELIVERY**

Do you use medical/dental services in Ukraine when you travel there on holiday? If yes: what are the benefits?

Do health care workers in Poland know enough about the Ukrainian health care and vaccine delivery system - do Ukrainians living in Poland have to explain the differences?

**TRUST IN STATE STRUCTURES**

Do the Ukrainian authorities generally support vaccination?

Are there enough vaccines for everyone in need in Ukraine?

What factors could affect the increase in the number of people vaccinated in Ukraine? Would subsidizing doctors for vaccinations increase the percentage of those vaccinated?

**TRUST IN QUALITY OF HEALTHCARE**

Do you think Ukrainians in Poland have confidence in Polish healthcare workers?

a. doctors

b. nurses / midwives

c. other employees

Do you think Ukrainians in Poland have confidence in Ukrainian healthcare workers?

a. doctors

b. nurses / midwives

c. other employees

**TRUST IN VACCINATION POLICIES & PROVISION**

Are you aware that vaccination schedules are slightly different in Poland and Ukraine?

Some vaccinations in Poland are compulsory, and others are not and you usually have to pay for them - do you think Ukrainians in Poland praise it as a good system? How do they perceive the Polish vaccination program in general?

What were the differences in your perception between Polish and Ukrainian vaccination systems?

Have you experienced the Polish vaccination system? Were there any good or bad sides in the way Polish and Ukrainian systems were implemented in your practice?

Have you heard more positive opinions about the vaccination system in Ukraine than about the system in Poland? Can one get a false vaccination certificate in Ukraine, in Poland?

**TRUST IN THE VACCINES PROVIDED (VACCINATION SAFETY/QUALITY/IMPORTANCE)**

Do you think that Ukrainians generally consider vaccinations safe?

Do you think that Ukrainians generally consider vaccinations effective?

How important is that:

a. children receive routine childhood immunizations (such as measles, mumps, rubella and others)

b. seasonal influenza vaccine is administered in school children/elderly people/ pregnant women

c. teenage girls/boys are vaccinated against HPV

d. vaccinations are current in pregnant women (smallpox/rubella/warts)
